# TPX2 enhances the transcription factor activation of PXR and enhances the resistance of hepatocellular carcinoma cells to antitumor drugs

**DOI:** 10.1038/s41419-022-05537-7

**Published:** 2023-01-27

**Authors:** Hongbo Wang, Fang Chu, Xiao-feng Zhang, Peng Zhang, Li-xin Li, Yun-long Zhuang, Xiao-feng Niu, Xi He, Zhi-jie Li, Ying Bai, Da Mao, Zhen-wen Liu, Da-li Zhang, Bo-an Li

**Affiliations:** 1 Senior Department of Hepatology, the Fifth Medical Center of Chinese People’s Liberation Army General Hospital, Beijing, 100039 China; 2 Department of Emergency, the Fifth Medical Center of Chinese People’s Liberation Army General Hospital, Beijing, 100039 China; 3 Department of Urology, Chinese People’s Liberation Army (PLA) General Hospital/Chinese PLA Medical Academy, Beijing, 100853 China; 4 Department of Nutrition and Food Hygiene, School of Public Health, Peking University, Beijing, 100191 China; 5 Division of Chemical Metrology and Analytical Science, National Institute of Metrology, Beijing, 100029 China; 6 Clinical Laboratory, the Fifth Medical Center of Chinese People’s Liberation Army General Hospital, Beijing, 100039 China

**Keywords:** Cancer metabolism, Experimental models of disease

## Abstract

The pregnane X receptor (PXR) is an important regulator of hepatocellular carcinoma cellular resistance to antitumor drugs. Activation of PXR was modulated by the co-regulators. The target protein for the Xenopus plus end-directed kinesin-like protein (Xklp2) known as TPX2 that was previously considered as a tubulin regulator, also functions as the regulator of some transcription factors and pro-oncogenes in human malignances. However, the actions of TPX2 on PXR and HCC cells are still unclear. In the present study, our results demonstrate that the high expression of endogenous mRNA level of TPX2 not only correlated with the poor prognosis of advanced HCC patients who received sorafenib treatment but also with expression of PXR’s downstream genes, *cyp3a4* and/or *mdr-1*. Results from luciferase and real-time polymerase chain reaction (qPCR) showed that TPX2 leads to enhancement of the transcription factor activation of PXR. Protein–protein interactions between PXR and TPX2 were identified using co-immunoprecipitation. Mechanically, overexpression of TPX2 led to enhancement of PXR recruitment to its downstream gene *cyp3a4*’s promoter region (the PXRE region) or enhancer region (the XREM region). Treatment of HCC cells with paclitaxel, a microtubule promoter, led to enhancement of the effects of TPX2, whereas vincristine, a microtubule depolymerizing agent caused a decrease in TPX2-associated effects. TPX2 was found to cause acceleration of the metabolism or clearance of sorafenib, a typical tyrosine kinase inhibitor (TKI) in HCC cells and in turn led to the resistance to sorafenib by HCC cells. By establishing novel actions of TXP2 on PXR in HCC cells, the results indicate that TPX2 could be considered a promising therapeutic target to enhance HCC cells sensitivity to antitumor drugs.

## Introduction

The pregnane X receptor (PXR) is a member of the nuclear receptor protein superfamily (nuclear receptor subfamily 1, group I, member 2 [NR1I2]), which is structurally characterized by DNA- and ligand-binding domains (the ligand-binding domain contains the transcription activation domain [the AF-2] of PXR) [1]. PXR is the regulatory hub for the body’s metabolism and clearance of exogenous drugs and toxic agents, and it can also participate in the regulation of hepatocellular carcinoma (HCC) and other malignant tumor cells’ resistance to anti-tumor drugs [2]. In HCC cells, PXR can be activated by its ligands/agonists after which the activated PXR can upregulate the expression of its downstream genes, including *cyp3a4* and *mdr-1*. This process leads to the acceleration of the metabolism and clearance rate of antitumor drugs and finally leads to induction of HCC cellular resistance to antitumor drugs [3, 4]. PXR activity is affected by co-regulators, and it is of great scientific and clinical significance to discover and identify novel transcriptional co-regulators of PXR in HCC cells [5, 6].

TPX2 is the target protein for Xenopus plus end-directed kinesin-like protein (Xklp2) and contains the TPX domain. It has previously been considered a micro-tubulin interacting protein and recently has been considered an important regulator of human malignancy proliferation and metastasis [7, 8]. Our previous publication and some other publications reported that TPX2 may also function as regulators of transcription factors (ETS-1) or nuclear receptors (androgen receptor [AR]) [9, 10]. However, the detailed role of TPX in HCC and specifically in HCC cellular resistance to antitumor drugs is largely unknown. In the present study, our results reveal a novel function for and mechanism of TPX2 enhancement of the metabolism and clearance of antitumor drugs by HCC cells and/or resistance of HCC cells to antitumor drugs via the role of TPX as a co-activator of PXR. Results not only extend our knowledge of the role of TPX2 in enhancing the resistance of HCC cells to antitumor drugs but also provides novel ideas for HCC treatment.

## Materials and Methods

### Clinical specimens related material and cell lines

The human-related experimental materials involved in this study consisted of mainly cDNA samples extracted from patient-derived HCC clinical specimens or the HCC cell lines. The cDNA samples were stored in a –80 °C refrigerator and stored in aliquots. Samples from a total of 52 patients with advanced HCC treated with sorafenib and paired para-cancerous tissues (the nontumor tissues) were obtained [11–13]. Baseline information was detailed in our previous publications [14, 15]. The cell lines use in this study included various HCC cell lines (HepG2 [a HCC cell line], MHCC97-H [a highly aggressive HCC cell line], and MHCC97-L [a lowly aggressive HCC cell line]) and a PXR positive colorectal cancer line LS180. These cells were purchased from the National Infrastructure of Cell Resources of the Chinese Academy of Medical Sciences/China Union Medical College and descripted in our previous work [16].

### Agents: vectors and antitumor drugs

#### Antitumor drugs

Antitumor drugs included tyrosine kinase inhibitor (TKIs) sorafenib, lenvatinib, regorafenib, and cabozantinib were synthesized by Professor and Dr. Cao Shuang at Wuhan Engineering University (Wuhan, China) and the purity of these drugs’ powders were > 99% according to high-performance liquid chromatography (HPLC) as shown in Table 1. Some cytotoxic chemotherapeutic agents, doxorubicin (Cat. No.: E2516), paclitaxel (Cat. No.: S1150), etoposide (Cat. No.: S1225), and irinotecan (Cat. No.: S1198), were purchased from Selleck Corporation, Houston, Texas, USA. For cellular experiments, the four TKIs, the three cytotoxic chemotherapeutic drugs, irinotecan, paclitaxel, and etoposide, and the pure powders of these seven drugs were first weighed accurately using a precision balance (1/100,000 precision required) and then dissolved in the organic solvent dimethylsulfoxide (DMSO). In the process of dissolving these pure drug powders, stirring, vortexing, and ultrasonic vibration were used to assist dissolution [17, 18]. After a drug was fully dissolved, Dulbecco’s modified Eagle’s medium (DMEM) without fetal bovine serum (FBS) was used to dilute the drug dissolved in DMSO. For doxorubicin, we used DMEM without FBS to dissolve the drug. When performing cell viability experiments (tetrazolium [MTT] assays), these drugs were diluted. Table 2 shows the serial concentration gradients of these drugs used for treating cultured cells.

**Table 1:** The purifies of TKIs used in the presence work.

TKIs	The purifies from HPLC
sorafenib	99.1 (%)
lenvatinib	99.4
regorafenib	99.2
cabozantinib	99.5

*HPLC* High-performance liquid chromatography

**Table 2:** The concentrations of antitumor drugs used in the MTT experiments.

Antitumor drugs		Concentrations (μmol/L)						
TKIs	sorafenib	10.000	3.000	1.000	0.300	0.100	0.030	0.010
	lenvatinib	10.000	3.000	1.000	0.300	0.100	0.030	0.010
	regorafenib	10.000	3.000	1.000	0.300	0.100	0.030	0.010
	cabozantinib	10.000	3.000	1.000	0.300	0.100	0.030	0.010
Chemotherapies	doxorubicin	1.000	0.300	0.100	0.030	0.010	0.003	0.001
	paclitaxel	0.300	0.100	0.030	0.010	0.003	0.001	0.000
	etoposide	1.000	0.300	0.100	0.030	0.010	0.003	0.001
	irinotecan	3.000	1.000	0.300	0.100	0.030	0.010	0.003

For the animal experiments, the pure powder of sorafenib was accurately weighed using a precision balance (1/100,000 precision required) and then dissolved in the organic solvent DMSO with PEG400 (polyethylene glycol 400) and Tween 80. In the process of dissolving these pure drug powders, stirring, vortexing, and ultrasonic vibration were used to assist dissolution. After the drug was fully dissolved, phosphate-buffered saline (PBS) was used to dilute the drug dissolved in the DMSO with PEG400 and Tween 80 to form the oral formulation of sorafenib. The final concentration of sorafenib in the formulation was about 0.5 mg/ml [19].

#### Vectors for transfection and luciferase assay

The expression vectors used in this study, mainly the expression vector with TPX2 and the expression vector with TPX2 and its small interfering RNA (siRNA), were provided by Professor and Dr. Peng Zhang in the Senior Department of Urology, the Third Medical Center of PLA (Chinese People’s Liberation Army) General Hospital, Beijing 100039, China as reported and described in our previous publications [9, 10]. The luciferase reporters were described in our previous publications [14, 15]. Briefly, the PXRE sequence in the −362/+52 region of the *cyp3a4*’s promoter region of the PXR downstream gene was cloned into the pGL3Promoter vector to form PXRE-Luc. The XREM sequence in the −7836/−7208 region of the PXR downstream gene cyp3a4’s enhancer region was cloned into the pGL3Basic vector to form XREM-Luc. The binding element of PXR in the cyp3a4 promoter and enhancer regions were DR3 and ER6, respectively, and the 5-mer DR3 or ER6 was cloned into the pGL3Promoter vector to form DR3-Luc or ER6-Luc [20].

#### The Vectors for the Immunoprecipitation

The FLAG tagged PXR (including full length sequence of PXR, NTD [N-terminal domain, residues 1–40], DBD [DNA-binding domain, residues 41–110], HD [hinge domain, residues 1–140] and LBD [ligand-binding domain, residues 141–434]) or HA tagged PXR [14]; FLAG tagged TPX2 (including 1–45aa, 46–140aa, 141–280aa, 281–320aa, and 321–747aa) or HA tagged TPX2 were cloned into the pcDNA 3.1 plasmids and subjected to immunoprecipitation experiments.

### Drug metabolism experiments

The metabolism and clearance rates of sorafenib were measured in cultured HCC cells or in subcutaneous tumor tissue formed by HCC cells, respectively [11, 12]. For cultured cell experiments, the cultures of HCC cells (MHCC97-H, MHCC97-L, or HepG2) were first transfected with the corresponding vector into the cells after which the HCC cells were incubated with 1 μmol/L sorafenib for about 12 h. After incubation with sorafenib was complete, the medium containing sorafenib was discarded and replaced with normal DMEM + FBS for continuous cultures. Cell samples were collected at each time point starting at 0 (when the cell samples were collected immediately after the incubation step/baseline) followed by 4, 8, 12, 24, and 48 h.

For HCC subcutaneous tumor tissue, HCC cells were first cultured for transfection after which the cells were inoculated subcutaneously into nude mice to form tumor tissue. When the tumor tissue volume reached about 1500 mm^3^, the sorafenib solution was prepared and the solution was directly injected into the tumor tissue. The HCC tumor tissues were collected at each time point described previously with the addition of 72 and 96 h.

For sorafenib-treated samples in cellular experiments, sorafenib processing and extraction of the samples were performed. HCC cell samples were re-suspended in PBS after which the organic solvent acetonitrile at a ratio of 1:1 was added. The samples were then crushed and extracted for about 30 min with vortex shaking. After that step, the samples were centrifuged at 12,000 rpm at 4 °C for about 15 to 20 min. At this time, the samples were separated into cell debris, organic, and water phases. The collected organic phase contained the sample of sorafenib in acetonitrile.

During the extraction of sorafenib samples from HCC tissue samples, the tissue samples were shredded to form tissue micro-blocks and then ground with liquid nitrogen. The samples ground with liquid nitrogen were mixed with PBS and then mixed with the organic solvent acetonitrile at a ratio of 1:1. The samples were then crushed and extracted for about 30 min using vortex shaking. After that, the samples were centrifuged at 12,000 rpm and at 4 °C for about 15 to 20 min. At this time, the sample was separated into cell debris and organic and water phases.

The collected organic phase contained the sample of sorafenib in acetonitrile, and the amount of sorafenib in these collected acetonitrile samples was determined using high-performance liquid chromatography–mass spectrometry/mass spectrometry (LC–MS/MS) [19, 20]. The specific conditions for the LC–MS/MS are shown in Table 3. S transition pair was detected using LC–MS/MS: (1) the parent ion was 465 Da and (2) the product ion was 252 Da.

**Table 3:** The Mass spectrometer settings to examine the amount of sorafenib in HCC cells or tissues by LC-MS/MS.

LC-MS/MS Parameters	Settings
Run duration (min)	10.0
The Ionspray voltage (kV)	3.0
The Sheath gas (N_2_) (psi)	35.0
Auxiliary gas (N_2_) (psi)	15.0
Ion sweep gas (N_2_) (psi)	2.0
Tube lens off-set (V)	12.0
Capillary temperature (°C)	350.0
Collision pressure (argon) (mTorr)	1.5
Chrom filter peak width (s)	10.0

For the blood concentration assay, mice were orally dosed with sorafenib at a dose of 2 mg/kg, and blood samples (approximately 100 μl volume of blood was collected at each time point) were collected from the tail vein at the 6 h, 12 h, 24 h and 48 h time points, respectively, and the plasma was separated by centrifugation, and the plasma was assayed for sorafenib using LC-MS/MS.

### The quantitative polymerase chain reaction

Using the quantitative real-time polymerase chain reaction (qPCR) to detect the expression levels (the mRNA level) of the corresponding factors in HCC cells or HCC tissues [13, 21]. The qPCR ran under specific conditions: (1) the cDNA samples derived from HCC tissues from patients, (2) the mRNA samples extracted from cultured HCC cells, and (3) the mRNA samples extracted from subcutaneous tumor tissues of nude mice formed by HCC cells. In the qPCR and the subsequent steps of the experiments (luciferase or chromatin immunoprecipitation [ChIP]), the agonist/ligand (rifampicin, Cat. No.: [S1764] from Selleck Corporation, Houston, Texas, USA) or antagonist (ketoconazole, Cat. No.: [S1353] from Selleck Corporation, Houston, Texas, USA) of PXR was also used. These two agents were dissolved in DMSO and diluted with phenol red-free DMEM without FBS (The final concentration of DMSO did not exceed 1%). The solvent control used in these experiments was phenol red-free DMEM containing 1% DMSO.

For cDNA samples, one-step qPCR experiments were performed directly using the Power SYBR^®^ Green RT-PCR Kit (Thermo Fisher, Waltham, MA, USA) on a 7500 series device (Applied Biosystems; Foster City, CA, USA). For cell samples, the lysis experiments were performed by using a lysis bead-vortex method after which RNA samples were reverse-transcribed to cDNA samples using the SuperScript™ IV VILO™ Reverse Transcription Kit. For HCC tissue samples, the lysate was separated using the liquid nitrogen trituration method prior to the reverse transcription experiments. The RNA samples extracted from tumor tissues were reverse-transcribed to cDNA using the SuperScript™ IV VILO™ Reverse Transcription Kit. Based on the previous experiments, the mRNA expression level of the target gene was detected by one-step qPCR. The relative expression level/mRNA level of a target gene was based on the ratio of its cycle-threshold value (ct values) to the cycle-threshold value of the loading control/internal reference (β-Actin). The primers used in the qPCR experiments are listed in Table 4. According to the method described in previous publications [22, 23], the qPCR results were plotted as a histogram, scatter plot, and heat map. Specifically, a heat map was drawn using the mean and standard deviation of the mRNA expression levels of the target genes for which the mRNA expression level of TPX2 in each HCC tissue specimen was taken as the abscissa, and the expression levels of PXR, retinoid X receptor (RXR), cyp3a4, and mdr-1 were used as the vertical axis to draw the scatter-plot images with the coordinates [22, 23]. The expression level of the control group was represented by “1”, and heat-map images of the folds of control of each group relative to the change of control were constructed [24].

**Table 4:** The primers used in the qPCR and ChIP assays in the presence work.

Genes	primers	sequences
cyp3a4	the forward primer	5’-CTAGCACATCATTTGGACTG-3
	the reverse primer	5’-ACAGAGCTTTGTGGGACT-3'
PXR	the forward primer	5’-AGAGCGGCATGAAGAAGGAGATG-3'
	the reverse primer	5’-GAAATGGGAGAAGGTAGTGTCAAAGG-3'
P-gp (mdr-1)	the forward primer	5’-CCATAGCTCGCGCCCTTGTTAGA-3'
	the reverse primer	5’-CGGTGAGCAATCACAATGCAG-3'
tpx2	the forward primer	5′-ACCTTGCCCTACTAAGATT-3′
	the reverse primer	5′-AATGTGGCACAGGTTGAGC-3′
β-Actin (loading control)	the forward primer	5’-CTCCATCCTGGCCTCGCTGT-3'
	the reverse primer	5’-GCTGTCACCTTCACCGTTCC-3'
RXR	the forward primer	5’-AGATGGACAAGACGGAGCTG-3’
	the reverse primer	5’-CCAAGGACGCATAGACCTTC-3’.
XREM	the forward primer	5’-TCTAGAGAGATGGTTCATTCC-3'
	the reverse primer	5’-TCTTCAACAGGTTAAAGGAG-3'
PXRE	the forward primer	5’-AGATCTGTAGGTGTGGCTTGTTGG-3'
	the reverse primer	5’-TGTTGCTCTTTGCTGGGCTATGTGC-3'
The Input	the forward primer	5’-AACCTATTAACTCACCCTTGT-3'
	the reverse primer	5’-CCTCCATTCAAAAGATCTTATTATTTAGCATCTCCT-3'

XREM (the −7836/−7208 region of the upstream sequences of *cyp3a4*’s transcription start site); PXRE (the −362/+52 region of the upstream sequences of *cyp3a4*’s transcription start site); The Input (non-specific genomic sequence)

### Luciferase assays

The HCC cells were co-transfected with plasmids (the TPX2 vectors or siTPX2 vectors with luciferase reporters). Cells were then treated with solvent control (DMEM without FBS with 1‰ DMSO) or rifampicin, a typical agonist of PXR (5 μmol/L dose of rifampicin diluted in DMEM and 1% DMSO without FBS). After the transfection experiment and the rifampicin treatment, cells were collected for luciferase and β-galactosidase activities, respectively, and the β-galactosidase activity was used to correct the results of the luciferase assay [25]. Results were expressed as relative luciferase activity of groups (fold of control). Results are displayed as a histogram of mean ± standard deviation.

### Immunoprecipitation, cell nucleoplasm separation and protein immunoblotting

For immunoprecipitation, after transfection with FLAG-PXR or FLAG-TPX2 in MHCC97-H cells, the cells were collected and fragmented by sonication (during which the cells were resuspended or fragmented using high salt IP buffer). Thereafter, FLAG-PXR/TPX2 complexes and FLAG-TPX2/PXR complexes were isolated from the system using FLAG-beads and detected using western blot. Further, HepG2 cells were co-transfected with a truncated mutant of FLAG-tagged series PXR and HA-tagged TPX2, or a truncated mutant of FLAG-tagged series TPX2 and HA-tagged PXR, respectively, after which FLAG-beads were used for sub-separation and detection. For nucleo-plasmic isolation/sub-fraction, after transfection of control or siTPX2 in MHCC97-H cells or control or TPX2 in MHCC97-L cells, the cells were treated with solvent control or Rifampicin, respectively, and then collected and fragmented using ultrasound, and the nuclei were isolated by centrifugation at 800 rpm for 3 min at 4 °C. The cytoplasmic fragments were separated by centrifugation at 12,000 rpm for 3 min at 4 °C. If no subcellular fraction was performed, the expression levels of TPX2, PXR, CYP3A4, P-GP, etc. were detected directly in the cell samples. These samples were detected by protein immunoblotting by subjecting FLAG-beads or cell samples to a boiling water bath for 15 min, followed by centrifugation at 12,000 rpm for 10 min at 4 °C. Thereafter, the supernatant samples were collected, and SDS-PAGE gels were configured according to the conventional method (for immunoprecipitation 15% SDS-PAGE gels were used), and sequentially The antibodies used in western blot experiments were classified as labeled antibodies (monoclonal antibodies to HA, FLAG), monoclonal antibodies to PXR, CYP3A4, P-GP, TPX2, etc., loading control (including β-Actin or Lamin A), which were purchased from Invitrogen (Thermo, USA).

### MTT-cell survival examination

The antitumor activity of antitumor drugs injuring HCC cells was examined by using MTT assay [26]. After culturing the HCC cells, transfection experiments were performed, and the cells were then seeded in 96-well cell culture plates (about 8000 cells per well). At the same time, various solutions containing antitumor drugs were prepared, and the drug solutions were added to the 96-well cell culture plate containing the HCC cells (Table 2 shows the doses of different antitumor drugs acting on HCC cells). After the cells were treated with the drug for about 48 h, a dose of 50 mmol/L tetrazolium (MTT) reagent was added, and cells with the MTT reagent were incubated for 5 to 6 h. After incubation with the MTT was complete, all liquid in the 96-well cell culture plate was discarded, and about 100 μl of DMSO was added to each well to dissolve the cell samples. During the process of lysing the cell samples, the cell culture plate was shaken for 15 minutes to completely lyse the cells. After the lysis step was complete, the cell culture plate was centrifuged at 12,000 rpm at 4 °C, and the supernatant was collected for evaluation [27].

### Chromatin co-immunoprecipitation

The vincristine (S9555) and rifampicin (S1764) used for chip were purchased from Selleck Company. Specifically, the pure pharmaceutical powder of vincristine or rifampicin was dissolved in the organic solvent DMSO and then diluted with phenol red-free DMEM without FBS. The recruitment of PXR to its downstream gene cyp3a4’s promoter or enhancer region was examined using ChIP assays. Experiments were performed according to the method described in the previous publications [28]. First, transfection experiments were carried out in HCC cells. After transfection, the cells were treated with different drugs (the PXR agonist rifampicin, the microtubule aggregation drug, paclitaxel, or the microtubule depolymerization agent, vincristine). After these transfection experiments and experiments with drug-treated cells, cell samples were collected for fixation, disruption, and cross-linking experiments. For the detection of DNA and protein complex samples, qPCR was used for detection to determine the amount of DNA sequences bound to the target protein. The target protein of the ChIP experiment in this study was PXR or RXR. The amplified sequences were the promoter and enhancer sequences (PXRE and XREM regions, respectively) of cyp3a4. The primers are listed in Table 4.

### In vivo tumor model

In the present work, the in vivo antitumor activation of TKI sorafenib and the effect of TPX2 on PXR or the sensitivity of HCC cells to sorafenib was examined by the subcutaneous tumor model [29]. The HCC cells were transfected with plasmids and then injected into the subcutaneous position of nude mice (the HepG2 cells were transfected with TPX2 for TPX2 overexpression and the siRNA of TPX2 for TPX2 knockdown). After the cells in the bottom of the dish were digested with trypsin, the cells were gently mixed with sterilized PBS to prepare a cell suspension. The resulting cell suspension was directly injected subcutaneously into nude mice using a pre-sterilized disposable medical syringe with a capacity of 1 ml. After that step, animal status was observed every day, and the tumor tissue was collected after 4 to 6 weeks of growth to detect the tumor volume (using a Vernier caliper to measure the long and short axes of the tumor tissue after which the tumor volume was calculated according to the formula: short axis × short axis × long axis/2) and weight (accurately weighed with a 1/10000th precision balance). Finally, the tumor tissue was ground with liquid nitrogen, and qPCR was then used to measure the mRNA expression level of the corresponding genes. The intrahepatic tumor model and the in vivo imaging of small-animals-related experiments were performed according to our previous publications [19, 20]. The hepG2 cells were transfected with plasmids and injected into nude mice to form subcutaneous tumors. The tumor tissues were prepared as micro-blocks, and the micro-blocks were directed transplanted into nude mice’s liver. The weights of the micro-block of the subcutaneous tumor tissues were listed as Table 5. The mice received sorafenib via oral administration. The in vivo imaging of small animals was performed using micro-positron emission tomography (^micro^PET).

**Table 5:** The weighs of HCC tissues’ micro-blocks for the intrahepatic transplantation experiment.

No.	control	Sorafenib	Sorafenib + TPX2	Sorafenib + siTPX2
	Weighs of HCC tissues’ micro-blocks (mg)			
1	1.80	1.37	2.04	1.77
2	1.49	2.33	1.62	1.32
3	1.67	1.97	1.99	1.83
4	1.83	1.82	1.47	1.85
5	2.15	1.31	1.45	1.95
6	1.70	1.64	1.67	1.59

### Ethics statement

In this study, acquisition, preservation and corresponding experimental protocols, experimental design, and experimental techniques of human-related experimental materials were reviewed and filed by the Medical Ethics Committee of the Fifth Medical Center of the Chinese People’s Liberation Army General Hospital. In this study, animal welfare and animal ethics related to the purchase, breeding, and experimental design of experimental animals were reviewed and filed by the Animal Ethics Committee of the Fifth Medical Center of the Chinese People’s Liberation Army General Hospital.

### Statistical analysis

All statistical analyses in the presence work were performed using the GraphPad 8.0 (GraphPad Software Corporation, Armonk, NY, USA). The half-maximal inhibitory concentration (*IC*_*50*_) of antitumor drugs or the half-life values of sorafenib in HCC cells or tumor tissues was calculated by using Origin 6.0 software (OriginLab Corporation, Northampton, Massachusetts, USA). For the between-group comparison for two datasets, the data were first evaluated in terms of normal distribution (according to the F value, if the F value is > 0.05, the data conformed to the normal distribution, and the unpaired t-test was used for testing; if the F value is < 0.05, the data did not conform to the normal distribution, and the rank sum test was used). For the survival analysis data, the survival curves were analyzed by the log-rank (Mantel–Cox) test to detect the P-value of the survival curve, the median survival time, and the 95% confidence interval (CI). For the co-relationship analysis, the data were analyzed by linear regression to determine the overall trend of a group of data points. *P* < 0.05 indicates that the slope of the regression equation was not 0, namely, a correlation between horizontal and vertical coordinates could be detected. If the slope was positive, the horizontal coordinate was positively related to the vertical coordinate, and if the slope was negative, the horizontal coordinate was negatively related to the vertical coordinate. For the multi-grouped data, the ordinary one-way analysis of variance (ANOVA) and multiple comparison methods were used. An ANOVA was used to examine whether any difference between groups could be detected. The multiple comparisons (t-test) was used to detect differences between pairs of data. The half-life values of sorafenib in HCC cell samples or HCC tumor tissues was calculated by the “best-fit values” “(Inhibitor) versus response–variable slope (four parameters)” methods. The results were expressed as t_1/2_ values with 95% CI values.

## Results

### TPX2 is associated with the poor prognosis of advanced HCC patients received sorafenib and the activation of PXR pathway

**Fig. 1:**
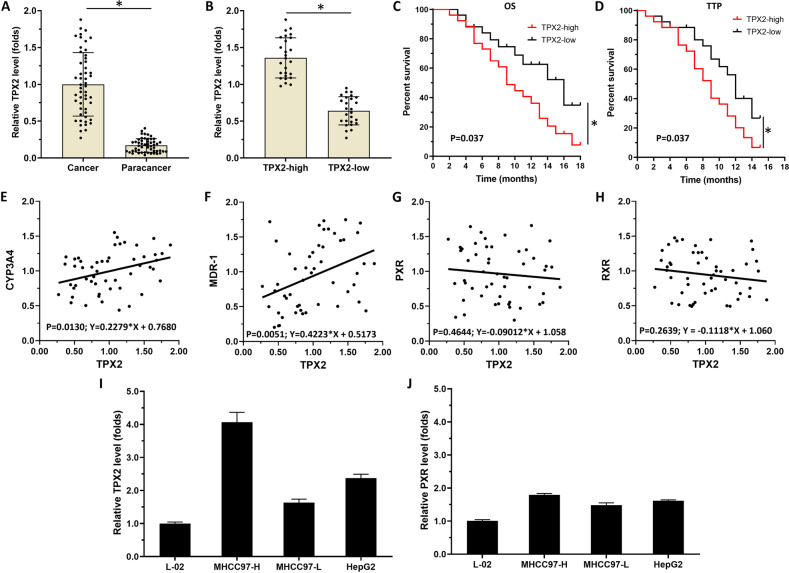
TPX2, which is the target protein for the Xenopus plus end-directed kinesin-like protein (Xklp2), associates with the poor prognosis of advanced hepatocellular carcinoma (HCC) patients received sorafenib treatment. TPX2 was found to be related to the pregnane X receptor (PXR) pathway in HCC clinical specimens. (**A** and **B**) the expression of TPX2 in HCC clinical specimens (**A** and **B**) and the paired non-tumor tissues were examined using real-time polymerase chain reaction (qPCR), and the advanced HCC patients were divided into two groups: (1) TPX2 high group and (2) TPX2 low group according to the median values of TPX2. (**C** and **D**) Survival curves of advanced HCC patients were obtained and compared with the patient grouping and patient clinical follow-up information. The results are shown as overall survival (OS) (**C**) or time to progression (TTP) (**D**). (**E**–**H**) the expression level of TPX2, *cyp3a4*, *mdr-1*, PXR, or retinoid X receptor (RXR) in HCC clinical specimens was examined by qPCR. After that, a scatter plot was drawn based on the results of qPCR with the expression level of TPX2 as the abscissa (**E**–**H**), and the expression levels of cyp3a4 (**E**), mdr-1 (**F**), PXR (**G**), and RXR (**H**) as the vertical axis. The coordinates were plotted as scatter plots, which were then analyzed using linear regression. The endogenous mRNA expression of TPX2 (**I**) or PXR (**J**) in the selected hepatic cell lines (L-02, MHCC97-H, MHCC97-L, or HepG2 cells) was examined using qPCR. *P < 0.05.

To explore the role of TPX2 in HCC, we first determined the clinical significance of TPX2 expression in HCC tissues. As shown in Fig. 1A, the expression level of TPX2’s mRNA was much higher in HCC clinical specimens when compared with the paired nontumor tissues. In HCC specimens (Fig. 1B), the patients were divided into two groups: (1) TPX2 high group and (2) TPX2 low group according to the sample’s median expression level. Combined with clinical follow-up data, survival curves of the two groups of patients were obtained: (1) TPX2 low group and (2) TPX2 high group. The prognosis of patients belonging to TPX2 low groups was much better compared with the TPX2 high group’s patients (Fig. 1C, D, respectively). The overall survival ([OS] 9.0 [6.6–11.4], month, median value 95% CI 16.0 [10.1–21.9], month, median value [95% CI], *P* = 0.037) or time-to-progression ([TTP] 95% CI 9.0 [7.1–10.9], month, median value [95% CI] versus 12.0 [10.5–13.5], month, median value [95% CI], *P* = 0.037) of the TPX2 low group was much longer when compared with the TPX2 high group (Table 6).

**Table 6:** The high level of TPX2 is associated with the poor prognosis of advanced HCC patients received sorafenib treatment.

Patients		TPX2 mRNA expression		P values
		High (*n* = 26)	Low (*n* = 26)	
OS	median value	9.0	16.0	0.037
	95% CI	6.6–11.4 (M)	10.1–21.9 (M)	
TTP	median value	9.0	12.0	0.037
	95% CI	7.1–10.9 (M)	10.5–13.5 (M)	

*TTP*’ time to progress, *OS* overall survival, *M*: months, *CI* confidence interval.

To further explore the roles of TPX2 in HCC, the relationship between TPX2 and the PXR pathway was examined. As shown in Fig. 1E–H, in HCC tissues, the expression level of TPX2 positively correlated with cyp3a4 and mdr-1 but not with PXR or RXR expression. Moreover, as shown in Fig. 1I, the expression level of TPX2 in HCC cells was significantly higher than that in the nontumor cells, L-02. Meanwhile, among the selected HCC cell lines, the expression level of TPX2 was the highest in MHCC97-H, the lowest in MHCC97-L cells, and HepG2 was between those two. The siRNA of TPX2 was used to knockdown TPX2 in MHCC97-H/HepG2 cells and overexpression of TPX2 was carried out in MHCC97-L/HepG2 cells. The expression of PXR in HCC cells was higher than that in L-02 cells, but only a small difference between several HCC cells was found (Fig. 1J). These results indicate that TPX2 is closely related to sorafenib resistance in HCC patients and is closely associated with the PXR pathway

### TPX2 leads to enhancement of the transcription factor activation of PXR in HCC cells

**Fig. 2:**
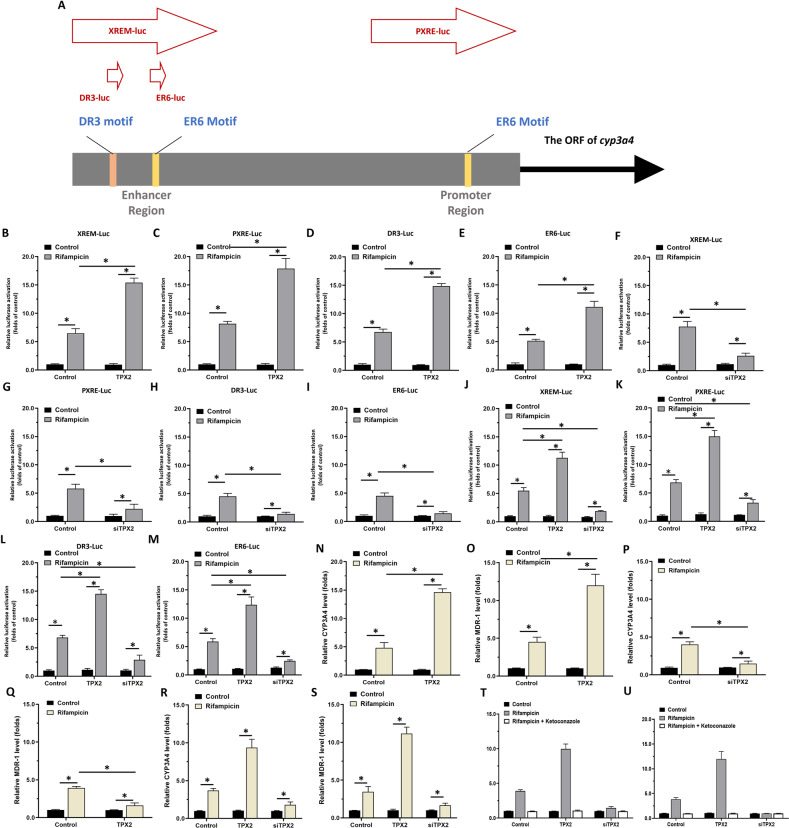
TPX2 enhances the transcription factor activation of ETS-1. (**A**) The downstream gene of PXR, *cyp3a4*, is upstream of the transcription initiation site, including the promoter region sequence (PXRE) and the enhancer region sequence (XREM). Two PXR binding sites (one DR3 motif and one ER6 motif) are found in XREM in addition to a PXR binding site (DR6 motif) in PXRE. (**A**) Schematic representation of the binding site of the cyp3a4 promoter to the enhancer region, PXR; schematic representation of the four luciferase reporters. The HCC cells, MHCC97-L (**B**–**E**), MHCC97-H (**F**–**I**), and HepG2 (**J**–**M**) were co-transfected with plasmids (control or TPX2 for MHCC97-L; control or siTPX2 for MHCC97-H; control, TPX2 or siTPX2 for HepG2 cells) (XREM-Luc [B, F and J]; PXRE-Luc [**C**, **G** and **K**]; DR3-Luc [**D**, **H** and **L**]; ER6-Luc [**E**, **I** and **M**]). Cells were treated with solvent control or rifampicin. The activation of XREM-Luc, PXRE-Luc, DR3-Luc, or ER6-Luc was examined by luciferase assays. (**N**-**U**) The HCC cells, MHCC97-L (**N** and **O**), MHCC97-H (**P** and **Q**) and HepG2 (**R** and **S**) were co-transfected with plasmids (control or TPX2 for MHCC97-L; control or siTPX2 for MHCC97-H; control, TPX2 or siTPX2 for HepG2 cells). Cells were treated with solvent control or rifampicin. The mRNA level of *cyp3a4* (**N**, **P** and **R)** or *mdr-1* (**O**, **Q** and **S**) was examined using qPCR. The effects of ketoconazole on TPX2 were shown (**T** and **U**). *P < 0.05.

To elucidate the potential effect of TPX2 on PXR, luciferase assays were first performed. As shown in Fig. 2, treatment with rifampicin, a typical agonist of PXR, induced the activation of the luciferase reporters (Fig. 2A), XREM-Luc, PXRE-Luc, DR3-Luc, and ER6-Luc (Fig. 2B–M) and in HCC cells (the MHCC97-L, MHCC97-H or HepG2 cells). Overexpression of TPX2 in MHCC97-L or HepG2 cells led to enhancement of the activation of luciferase reporters in the presence of rifampicin (Fig. 2B–E; J–M), whereas knockdown of TPX2 in MHCC97-H or HepG2 cells led to a decrease in the activation of luciferase reporters in the presence of rifampicin (Fig. 2F–I; J–M).

**Fig. 3:**
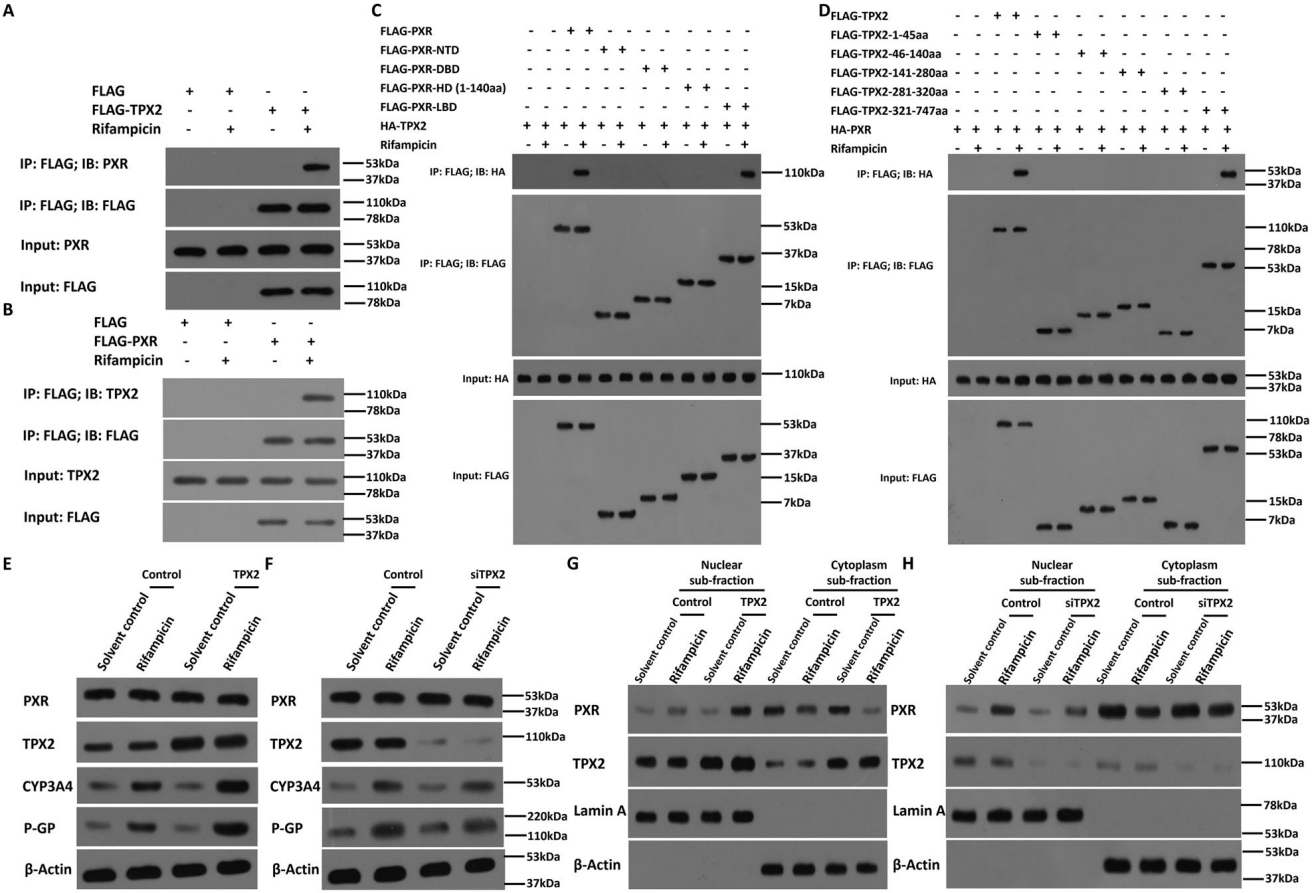
TPX2 interacts with PXR and affect the nuclear accumulation of PXR in HCC cells. (**A** and **B**) The interaction between TPX2 and PXR was identified by “IP: FLAG; IB: PXR” or “IP: FLAG; IB: TPX2”. (**C** and **D**) The interaction between FLAG-PXR mutation (including NTD [N-terminal domain, residues 1–40], DBD [DNA-binding domain, residues 41–110], HD [hinge domain, residues 1–140] and LBD [ligand-binding domain, residues 141–434]), and HA-TPX2; FLAG-TPX2 mutations (including 1–45aa, 46–140aa, 141–280aa, 281–320aa, and 321–747aa) and HA-PXR was identified by “IP: FLAG; IB: HA”. (**E** and **F**) The HCC cells, MHCC97-L (**E**), MHCC97-H (**F**), were co-transfected with plasmids (control or TPX2 for MHCC97-L; control or siTPX2 for MHCC97-H). Cells were treated with solvent control or rifampicin. The protein level of CYP3A4 or P-GP was examined using western blot. (**G** and **H**) The HCC cells, MHCC97-L (**E**), MHCC97-H (**F**), were co-transfected with plasmids (control or TPX2 for MHCC97-L; control or siTPX2 for MHCC97-H). Cells were treated with solvent control or rifampicin. The cells were separated into nuclear sub-fraction or cytoplasm sub-fraction. The protein level of PXR or TPX2 was examined using western blot.

Next, the effects of TPX2 on two downstream genes of PXR, *cyp3a4* and *mdr-1*, was examined using qPCR in HCC cells. As shown in Fig. 2N–S, the treatment of rifampicin induced the mRNA level of *cyp3a4* and *mdr-1* in HCC cells. Overexpression of TPX2 in MHCC97-L or HepG2 cells caused an enhancement of the mRNA level of *cyp3a4* or *mdr-1* in the presence of rifampicin (Fig. 2N–S), whereas knockdown of TPX2 in MHCC97-H or HepG2 cells led to a decrease in the mRNA level of *cyp3a4* or *mdr-1* in the presence of rifampicin (Fig. 2N–S). The antagonist of PXR, ketoconazole, almost blocked the effects of rifampicin and TPX2 on PXR’s downstream gene transcription (mRNA levels) as shown in Fig. 2T, U. Similar results were obtained by the protein level of CYP3A4 or P-GP (encoded by mdr-1) by western blot (Fig. 3E, F). Therefore, TPX2 causes enhancement of transcription factor activation of PXR in a ligand-dependent manner.

### TPX2 interacts with PXR in HCC cells

To explore the potential action of TPX2 on PXR, the potential protein interactions between PXR and TPX2 were examined using co-immunoprecipitation in HCC cells. As shown in Fig. 3A, FLAG-TPX2 interacted with PXR in the presence of rifampicin. Similar results were obtained in the re-IP experiments in which FLAG-PXR interacted with TPX2 in the presence of rifampicin (Fig. 3B). Further, the binding regions in PXR and TPX2 were examined. HepG2 cells were co-transfected with FLAG-tagged truncated PXR vectors with HA-TPX2 or FLAG-tagged truncated TPX2 vectors with HA-PXR (Fig. 3C, D) and cells were harvested for Co-IP experiments. Results in Fig. 3C, D demonstrates that HA-TPX2 interacted with the LBD 0 f PXR in a ligand-dependent manner (in the presence of rifampicin). HA-PXR interacted with the 321–747aa region of TPX2.

Next, the nuclear accumulation of PXR affected by TPX2 was assessed. As shown in Fig. 3G, H, PXR was distributed in both the nucleus and cytoplasm of MHCC97-H or MHCC97-L cells in the quiescent state and could translocate from the cytoplasm into the nucleus in the presence of its agonist: rifampicin. Overexpression of TPX2 enhanced the accumulation of PXR in the nucleus of MHCC97-L cells in the presence of rifampicin (Fig. 3G), whereas knockdown of TPX2 decreased the PXR nuclear accumulation in the presence of rifampicin (Fig. 3H). Therefore, TPX2 could interact with PXR in HCC cells in a ligand-dependent manner.

### TPX2 enhances the recruitment of PXR to its downstream gene cyp3a4’s promoter or enhancer regions

**Fig. 4:**
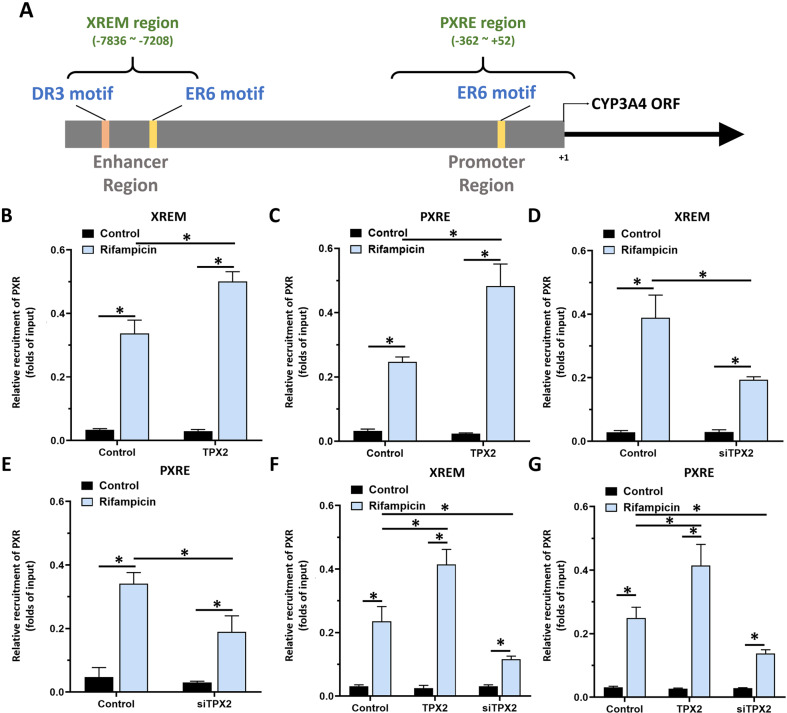
TPX2 enhances the recruitment of PXR to its downstream gene *cyp3a4*’s promoter or enhancer regions. (**A**) The downstream gene of PXR, *cyp3a4*, is upstream of the transcription initiation site, including the promoter region sequence (PXRE) and the enhancer region sequence (XREM). Two PXR binding sites (one DR3 motif and one ER6 motif) are located in XREM in addition to a PXR binding site (DR6 motif) in PXRE. In chromatin co-immunoprecipitation (ChIP), XREM and PXRE sequences were amplified respectively. The HCC cells, MHCC97-L (**B** and **C**), MHCC97-H (**D** and **E**) and HepG2 (**F** and **G**), were transfected with plasmids (control or TPX2 for MHCC97-L; control or siTPX2 for MHCC97-H; control, TPX2 or siTPX2 for HepG2 cells). Cells were treated with solvent control or rifampicin and analyzed using ChIP. *P* < 0.05.

The next step experiments were performed to reveal whether TPX2 could cause enhancement of the recruitment of PXR to its downstream gene *cyp3a4*’s promoter or enhancer region. As shown in Fig. 4, treatment with a 5 μmol/L dose of rifampicin induced the recruitment of PXR in the promoter (PXRE region) or enhancer (XREM region) region of *cyp3a4*. Overexpression of TXP2 in MHCC97-L cells led to enhancement of the recruitment of PXR to its downstream gene’s enhancer or promoter region in a ligand-dependent manner (Fig. 4A–C, F, G), whereas knockdown of TPX2 *via* its siRNA in MHCC97-H or HepG2 cells (Fig. 4A, D–G). Therefore, TPX2 appears to lead to enhancement of PXR recruitment to its downstream gene *cyp3a4*’s promoter or enhancer regions in a ligand-dependent manner.

### TPX2 accelerates the metabolism or clearance of sorafenib in cultured HCC cells or HCC tumor tissues

**Fig. 5:**
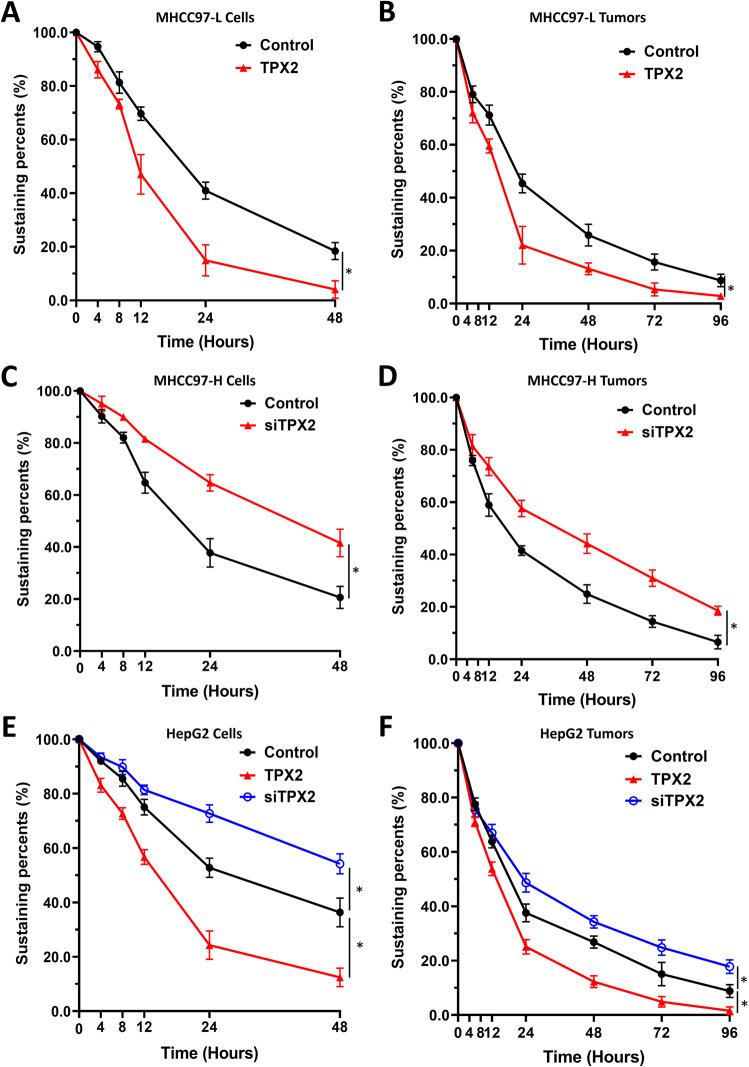
TPX2 promotes the metabolism or clearance of sorafenib in HCC cells or tumors. HCC cells MHCC97-L (**A** and **B**), MHCC97-H (**C** and **D**) or HepG2 (**E** and **F**) cells were cultured. The experiments were performed in cultured cells (**A, C** and **E**) or subcutaneous tumor tissues formed by HCC cells (**B**, **D** and **F**). The amount of sorafenib in cell or tumor samples was examined using liquid chromatography–mass spectrometry (LC–MS/MS). A retention curve of sorafenib was constructed according to the content of sorafenib measured in the samples at the 0 time point (baseline) and the content of sorafenib measured in the samples at each time point. *P < 0.05.

Next, whether the effects of TPX2 cause acceleration of a the metabolism and/or clearance of sorafenib, a standard TKI for advanced HCC treatment in HCC cells or HCC tumor tissues were examined by LC–MS/MS. As shown in Fig. 5, sorafenib was gradually metabolized and eliminated in cultured HCC cells, and at the 48-h time point, sorafenib was almost completely metabolized and eliminated in HCC cells. In MHCC97-L and HepG2 cells, overexpression of TPX2 caused the acceleration of the sorafenib metabolism or clearance (Fig. 5A, E), and the half-life (t_1/2_) value of sorafenib in HCC cells decreased (Table 7). Moreover, the knockdown of TPX2 by its siRNA in MHCC97-H or HepG2 cells led to the deceleration of sorafenib metabolism or clearance (Fig. 5C, E), and an increase in the t_1/2_ value of sorafenib (Table 7).

**Table 7:** Overexpression of TPX2 accelerates and knockdown of TPX2 decelerates the metabolism or clearance of sorafenib in HCC cells and tumor tissues.

Cell lines		Control	TPX2	siTPX2
		t_1/2_ values (hours)		
MHCC97-L	cultured cells	18.68 (16.52–22.13)	11.92 (10.86–13.34)	N.A.
	tumor tissues	28.32 (21.99–44.21)	13.49 (11.74–15.96)	N.A.
MHCC97-H	cultured cells	15.55 (13.62–18.87)	N.A.	36.61 (24.00–131.2)
	tumor tissues	19.67 (13.4–42.24)	N.A.	27.49 (20.44–45.80)
HepG2	cultured cells	18.86 (15.65–26.51)	13.70 (11.99 to 16.71)	>24 h
	tumor tissues	29.41 (16.01–37.56)	18.51 (12.29–24.83)	42.24 (25.08–137.2)

t_1/2_ values (hours) were shown as median values (95% confidence interval)

Next, sorafenib metabolism or clearance in HCC subcutaneous tumor tissues was also examined. As shown in Fig. 5, sorafenib was gradually metabolized and eliminated in HCC tissues, and at the 72-h time point, sorafenib was almost completely metabolized and eliminated in HCC tissues. In HCC tumors formed by MHCC97-L cells or HepG2, overexpression of TPX2 led to the acceleration of the metabolism or clearance of sorafenib (Fig. 5B, F), and the t_1/2_ value of sorafenib decreased (Table 7). Moreover, knockdown of TPX2 via its siRNA in MHCC97-H cells led to a deceleration of sorafenib metabolism and/or clearance in HCC tissues (Fig. 5D, F) and an increase in the t_1/2_ value of sorafenib (Table 7). Therefore, TPX2 appears to cause an acceleration of the metabolism and/or clearance of sorafenib in HCC cells or HCC tumor tissues.

### TPX2 promotes the resistance of HCC cells to antitumor drugs

**Fig. 6:**
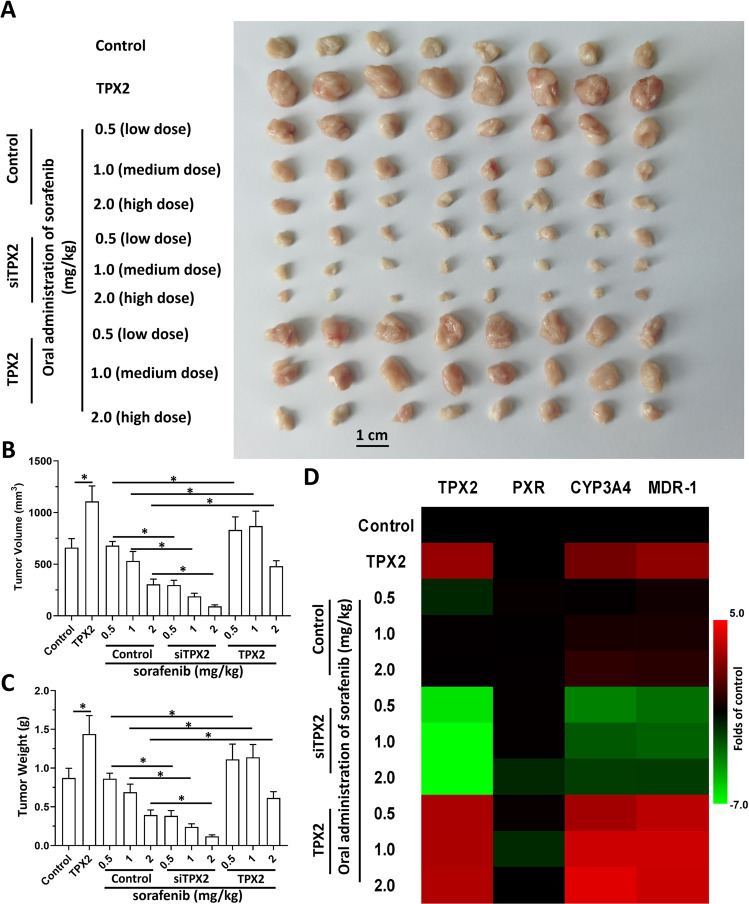
TPX2 promotes the resistance of HCC cells to sorafenib in a subcutaneous tumor model. The HepG2 cells were transfected with control, TPX2, or siTPX2. Cells were then injected into the subcutaneous position of nude mice. The mice received 2.0 mg/kg (high dose), 1.0 mg/kg (medium dose) or 0.5 mg/kg (low dose) of sorafenib via oral administration. After treatment, the tumors were collected to measure the tumor volumes, tumor weights, and/or the mRNA level of genes (TPX2, PXR, *cyp3a4* or *mdr-1*) by qPCR. The results were shown as images of subcutaneous tumors (**A**), histograms of tumor volumes/weights (**B** and **C**) or the heat-map of the mRNA level of genes (TPX2, PXR, *cyp3a4*, or *mdr-1*). *P < 0.05.

**Fig. 7:**
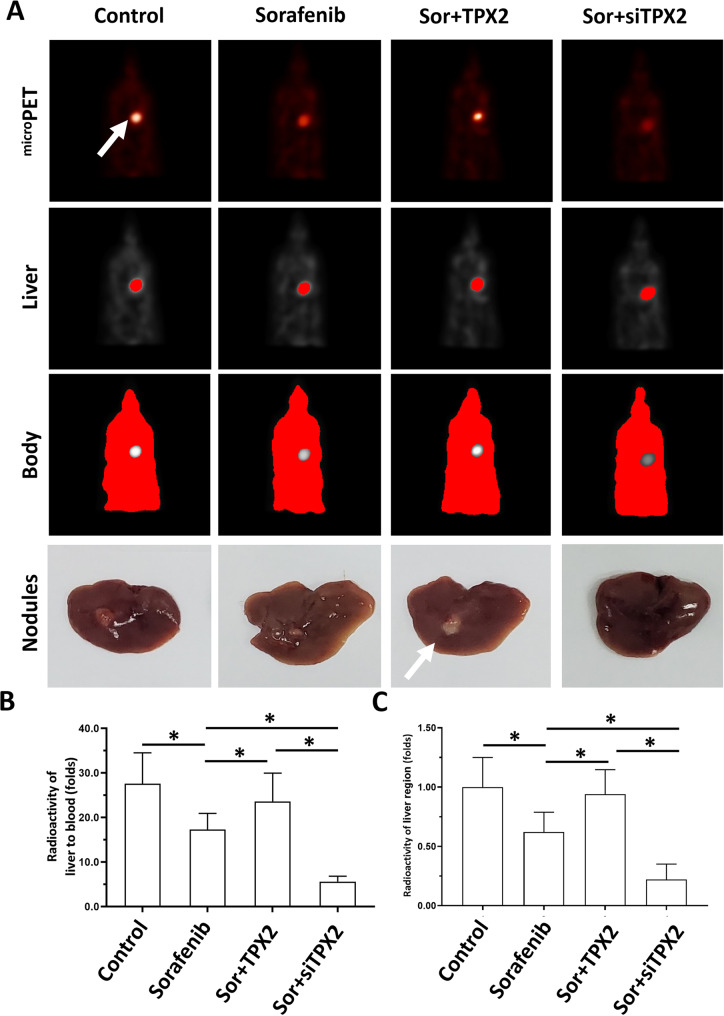
TPX2 promotes the resistance of HCC cells to sorafenib in an intrahepatic subcutaneous tumor model. The HepG2 cells were transfected with control, TPX2 or siTPX2 and injected into the subcutaneous position of nude mice. The tumor tissues were prepared as the micro-blocks for constructing the intrahepatic HCC model. The mice received the 1.0 mg/kg concentration of sorafenib via oral administration. The results were shown as images of micro positron emission tomography (microPET) (**A**) or quantitative results. *P < 0.05.

Further experiments examined whether TPX2 could modulate the sensitivity of HCC cells to antitumor drugs. The results are shown as Tables 8–10. Overexpression of TPX2 in MHCC97-L or HepG2 cells could induce the resistance of HCC cells to TKIs as reflected by an increase in *IC*_*50*_ values. Moreover, the knockdown of TPX2 in either MHCC97-H or HepG2 cells could enhance the sensitivity of HCC cells to TKIs, and the *IC*_*50*_ values of these drugs decreased. Similar results were also obtained from the TKIs and cytotoxic chemotherapeutic agents (Tables 8–10). TPX2 promotes the resistance of HCC cells to antitumor drugs. Moreover, in subcutaneous tumor models, overexpression of TPX2 promoted the subcutaneous growth of HepG2 cells and sorafenib inhibited the subcutaneous growth of HepG2 cells in a dose-dependent manner (Fig. 6A–C). Overexpression of TPX2 caused a decrease in the antitumor activation of sorafenib in HepG2 cells, whereas knockdown of TPX2 leads to enhancement of HepG2 cell sensitivity to sorafenib (Fig. 6A–C). Moreover, the effect of TPX2 on PXR pathway-related factors was examined with qPCR in subcutaneous tumor tissues (Fig. 6D). Similar results were also obtained from the intrahepatic HCC model in which HepG2 could form intrahepatic lesions in the liver organs of nude mice. Overexpression of TPX2 caused a decrease in the antitumor activation of sorafenib on HepG2 cells’ intrahepatic growth, whereas knockdown of TPX2 led to enhancement of the sensitivity of HepG2 cells to sorafenib (Fig. 7). Therefore, TPX2 appears to promote the resistance of HCC cells to antitumor drugs.

**Table 8:** Overexpression of TPX2 promotes the resistance of MHCC97-L cells to antitumor drugs, TKIs and chemotherapies.

Antitumor drugs	Control	TPX2
	*IC*_*50*_ values (μmol/L)	
sorafenib	1.86 (1.30–1.98)	4.84 (2.70–5.94)
lenvatinib	1.94 (1.85–2.36)	5.73 (4.89–6.17)
regorafenib	1.50 (1.10–1.61)	4.93 (4.55–5.26)
cabozantinib	1.47 (1.01–1.93)	5.03 (4.99–5.31)
doxorubicin	0.25 (0.10–0.33)	0.82 (0.60–1.05)
paclitaxel	22 (13.1–36.5) (nmol/L)	0.15 (0.04–0.33)
etoposide	0.38 (0.20–0.51)	1.32 (0.99–1.80)
irinotecan	0.43 (0.25–0.68)	0.97 (0.80–1.11)

*IC*_*50*_ values (μmol/L) were shown as median values (95% confidence interval)

**Table 9:** Knockdown of TPX2 enhances the sensitiivty of MHCC97-H cells to antitumor drugs, TKIs and chemotherapies.

Antitumor drugs	Control	siTPX2
	*IC*_*50*_ values (μmol/L)	
sorafenib	1.16 (0.95–1.31)	0.47 (0.31–0.74)
lenvatinib	1.07 (0.74–1.22)	0.50 (0.39–0.70)
regorafenib	0.99 (0.89–1.24)	0.75 (0.69–1.11)
cabozantinib	0.76 (0.66–0.97)	0.26 (0.15–0.46)
doxorubicin	0.20 (0.05–0.36)	0.06 (0.05–0.09)
paclitaxel	12.67 (6.5–17.3) (nmol/L)	4.20 (4.06–4.35) (nmol/L)
etoposide	0.25 (0.15–0.50)	0.10 (0.09–0.13)
irinotecan	0.52 (0.29–0.88)	0.16 (0.12–0.20)

*IC*_*50*_ values (μmol/L) were shown as median values (95% confidence interval)

**Table 10:** Overexpression of TPX2 promotes the resistance and knockdown of TPX2 enhances the sensitivity of HepG2 cells to antitumor drugs, TKIs and chemotherapies.

Antitumor drugs	Control	TPX2	siTPX2
	*IC*_*50*_ values (μmol/L)		
sorafenib	1.27 (0.82–1.75)	4.68 (3.90–4.83)	0.50 (0.34–0.58)
lenvatinib	1.30 (0.75–1.63)	5.89 (5.66–6.71)	0.33 (0.20–0.60)
regorafenib	1.01 (0.66–1.26)	3.65 (3.10–3.84)	0.62 (0.45–0.78)
cabozantinib	0.88(0.73–1.35)	4.78 (4.21–4.99)	0.39 (0.30–0.59)
doxorubicin	0.33 (0.06–0.48)	1.59 (1.36–1.80)	0.05 (0.03–0.07)
paclitaxel	14.78 (10.12–18.33) (nmol/L)	0.24 (0.18–0.37) (μmol/L)	8.31 (5.00–9.25) (nmol/L)
etoposide	0.46 (0.27–0.56)	1.93 (1.76–2.28)	0.18 (0.09–0.23)
irinotecan	0.30 (0.28–0.40)	0.82(0.73–0.93)	0.10 (0.08–0.15)

*IC*_*50*_ values (μmol/L) were shown as median values (95% confidence interval)

### TPX2 functions through tubulin

**Fig. 8:**
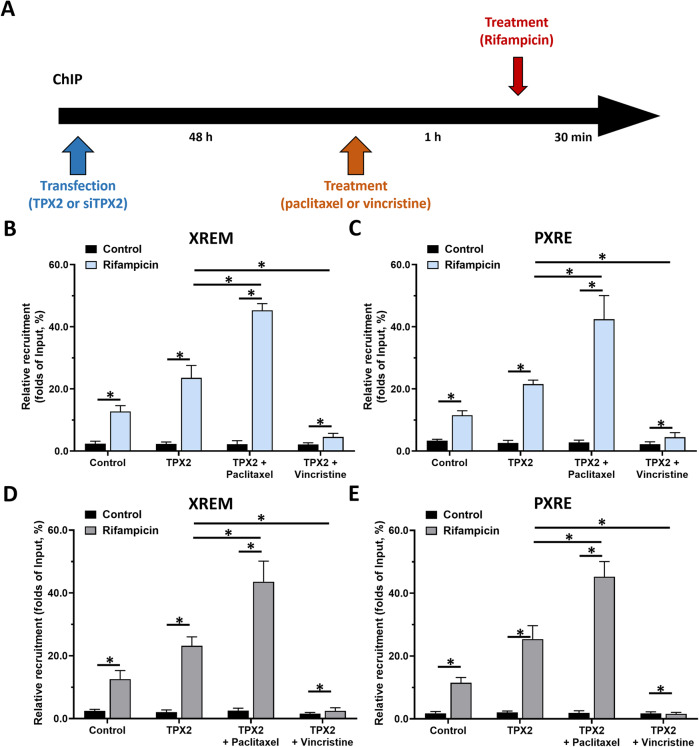
TPX2 promotes the recruitment of PXR to its downstream gene *cyp3a4*’s promoter or enhancer region. Rifampicin was used as an agonist of PXR, and the microtubule aggregation agent, paclitaxel, and the microtubule depolymerizing agent, vincristine, were used in combination with rifampicin. (**A**) The time points for this step are shown. Using a ChIP assay to detect the recruitment of PXR (**B** and **C**) or RXR in the enhancer (XREM) (**B** and **D**) and promoter (PXRE) (**C** and **E**) regions of *cyp3a4*. *P < 0.05.

Whether the effects of TPX2 on PXR are related to tubulin was examined by using paclitaxel or vincristine. As shown in Fig. 8, treatment with paclitaxel produced enhancement of the effect of TPX2 on PXR recruitment to *cyp3a4*’s promoter or enhancer regions. Treatment with vincristine almost blocked not only the effect of TPX2, but also the effect of rifampicin on PXR recruitment to cyp3a4’s promoter or enhancer (Fig. 8). Therefore, TPX2 most likely functions through tubulin’s function and integrity. Similar results were obtained with RXR (Fig. 8). These results further confirm the effect of TPX2 on the PXR pathway.

### TPX2’s functions in LS-180 cells

The above results were about HCC and that the liver is the main site of drug (including sorafenib) metabolism, the data in LS-180, a PXR positive colorectal cancer cell line, was also used. Overexpression or knockdown of TPX2 in PXR-positive colorectal cancer cells LS180 could affect PXR activity in LS180 cells (Supplemental Fig. 1). Overexpression of TPX2 in LS180 cells also promoted the metabolism and clearance of sorafenib, the t_1/2_ values decreased from 21.30 (16.21–25.37) to 12.04 (9.81–15.42); whereas knockdown of TPX2 increased the t_1/2_ values (hours) of sorafenib in LS180 cells (from 21.78 [18.37–23.60] to 32.67 [25.21–36.92]). Also, TPX2 overexpression or knockdown was able to affect the drug resistance of LS180 to sorafenib. The *IC*_*50*_ values (μmol/L) of sorafenib on LS180 cells were 0.91 (0.75–1.22) (control group), 4.30 (3.65–4.83) (TPX2 overexpression) or 0.25 (0.11–0.36) (TPX2 knockdown), respectively. These results further support the effect of TPX2 on PXR in HCC cells.

### Effect of Rifampicin on the metabolism of sorafenib in HCC tissues and the corresponding detection of sorafenib levels in plasma

The above used of rifampicin as an inducer of PXR and TPX in in vitro experiment. The data mentioning rifampicin as one of the intervention group in vivo were added. The results showed that, overexpression of TPX2 in MHCC97-L cells accelerated the metabolism and clearance of Sorafenib in the subcutaneous tumour tissues formed by HCC cells (the t_1/2_ values: 29.70 [21.38–36.75] for control group *v.s*. 14.01 [12.55–17.63] for TPX2 overexpression group), and oral administration of rifampicin at a dose of 5 mg/kg to mice further upregulated the effect of TPX2 (the t_1/2_ values: 14.01 [12.55–17.63] for TPX2 overexpression group *v.s*. 11.36 [8.10–14.03] for TPX2 overexpression + rifampicin group) to a certain extent.

Next, the above half-life values and clearance curves were measure in vivo: in the explanted tumours. The results in plasma and the oral route of administration was also shown as Supplemental Tables 1, 2. In nude mice, subcutaneous tumour tissues formed by TPX2 overexpressed HCC cells was able to reduce blood levels (plasma concentration) after sorafenib oral administration at a series of time points (2nd, 8th, 20th, 40th h), whereas in nude mice, intrahepatic tumour tissue formed by TPX2 overexpressed HCC cells was not able to affect blood levels after sorafenib oral administration at a series of time points (2nd, 8th, 20th, 40th h) (Supplemental Tables 1, 2). Moreover, in nude mice, subcutaneous tumour tissues formed by TPX2 knockdown HCC cells was able to increase blood levels (plasma concentration) after sorafenib oral administration at a series of time points (2nd, 8th, 20th, 40th h), whereas in nude mice, intrahepatic tumour tissue formed by TPX2 knockdown HCC cells was not able to affect blood levels after sorafenib oral administration at a series of time points (2nd, 8th, 20th, 40th h) (Supplemental Tables 1, 2). These results can be considered as supporting evidence.

## Discussion

Currently, the main strategy of anti-tumor drug treatment for HCC is still the use of molecular targeted therapy, and one of the most important strategies in this type of therapy occurs via various TKIs (tyrosine kinase inhibitors) [30]. These TKIs can inhibit the proliferation, metastasis, and invasion of HCC cells, and/or tumor angiogenesis [31–34]. Nevertheless, the antitumor effects of TKIs are unsatisfactory, and patients are also prone to develop resistance to TKIs [35, 36]. It has been clearly reported that only a small proportion (20%–40%) of patients with advanced HCC are considered sensitive to the TKIs sorafenib, but these patients’ disease has often progressed after sorafenib therapy (the secondary/acquired resistance) [37, 38]. Although no recognized and reliable indicator molecule for the prognosis of sorafenib treatment exists, much progress has been made in the research on the molecular mechanism of sorafenib treatment resistance [37, 38]. At present, it is generally believed that unlike NSCLC, mutations in TKIs (such as sorafenib) targets, which include receptor tyrosine kinases (RTKs, such as vascular endothelial growth factor receptor [VEGFR], platelet-derived growth factor [PDGFR], or c-kit or the kinase associated with the mitogen-activated protein kinase/phosphoinositide 3 kinase protein kinase B [MAPK/PI3K-AKT pathway]) in HCC cells are not the main mechanism for the difference in sensitivity or resistance to sorafenib in HCC patients [37, 38]. Correspondingly, various mechanisms of HCC cell resistance to sorafenib include several possibilities: (1) Notch, mammalian target of rapamycin [mTOR], and other cell pro-survival, anti-apoptosis-related pathways can lead to up-regulation of cell resistance to TKIs [39]; (2) the epithelial–mesenchymal transition (EMT) process can reduce the polarity of HCC cells and induce the resistance of HCC cells to sorafenib and other TKIs [40–42]; (3) HCC-related cancer stem cells and HCC cells are closely associated with sorafenib resistance [43]; and (4) the mutual compensation between different signaling pathways (such as c-MET can induce resistance to multiple TKIs, including sorafenib) [44]. Our group has conducted many studies on the resistance of HCC to molecularly targeted drugs and found that the PXR can also be an important mechanism for the resistance of HCC cells to sorafenib [19, 20]. Sorafenib can act as either a ligand or agonist of PXR to induce the transcription factor activity of PXR, and then induce its own drug resistance in a manner similar to negative feedback regulation by up-regulating the expression of downstream drug resistance genes of PXR [19, 20]. In addition to TKIs, antitumor drugs, such as paclitaxel, can also act as ligands for PXR and induce its activity [45]. Since the liver is the core organ for detoxifying (metabolism or clearance) the exogenous drugs and toxins, HCC cells derived from normal liver cells can be continuously stimulated by drugs during long-term treatment. PXR is activated [19, 20] and exerts a protective effect on HCC cells themselves by causing an increase in the resistance of HCC cells to antitumor drugs. Therefore, PXR may be considered a specific mechanism of HCC resistance to antitumor drugs and is of great significance for conducting PXR–HCC-related research.

In addition to the interaction of PXR with its ligands, transcriptional co-regulators of PXR are also important regulators of HCC resistance to various antitumor drugs [46, 47]. Early reports mainly focused on transcriptional activating cofactors, such as soluble complement receptor types 1 or 3 (SCR-1/3) or transcriptional repression cofactors, such as nuclear receptor co-repressor (NCoR) and silencing mediator of retinoic acid and thyroid hormone receptor (SMRT) [48]. Recent studies have shown that many proteins, including LINE-1 ORF-1p, can become novel transcriptional regulators of PXR, a finding that not only expands our understanding of PXR-related research but also forms the basis of the crosstalk between PXR and other signaling pathways [49]. Hypoxia-inducible factor EPAS-1 can also act as a positive regulator of PXR and lead to upregulation of the activity of the PXR pathway [50], which links the hypoxia mechanism to drug metabolism. miR-3609 can downregulate PXR activity and reverse HCC in cells by targeting EPAS-1 resistance to anticancer drugs [51]. The previous work from our group found that both transcription factors, ETS-1 and MTBP, can act as transcriptional activators of PXR, lead to up-regulation of the transcription factor activity of PXR and up-regulation of the expression levels of PXR downstream genes, CYP3A4 and MDR-1 [19, 20]. In the presence work, TPX2 could interact with PXR and affect the accumulation of PXR in nuclear or the recruitment of PXR to *cyp3a4*’s promoter or enhancer. The TPX2 could interact with the LBD region of PXR and PXR could interact with the 321–747aa region of TPX2. The LBD of PXR is consistent with the two interactions being ligand-dependent, while the 321–747aa region is the region where TPX2 is closely associated with microtubules. In terms of the downstream genes of PXR, CYP3A4 mediates phase I metabolism (oxidative metabolism) of antitumor drugs, and MDR-1 mediates phase III metabolism (transport and clearance of drugs) of antitumor drugs [52]. This process forms the basis of the body’s metabolism and clearance of exogenous drugs and toxicants under physiological conditions, but their function in HCC cells is that of drug-resistance genes [52]. In this study, after overexpression or knockdown of TPX2 in HCC cells, we could clearly observe that TPX2 led to up-regulation of the expression of PXR downstream drug resistance genes, *cyp3a4* and *mdr-1*. Our results also directly indicate that TPX2 overexpression can also promote the metabolism and clearance rates of sorafenib in HCC cells or tissues. Such results directly confirm the roles of TPX2 and PXR in HCC. Moreover, overexpression or knockdown of TPX2 in HCC cells basically affects the metabolism or clearance PXR in HCC cells, not the overall mice organism. HCC cells overexpressed or knockdown of TPX2 forming subcutaneous tumors are able to affect the blood concentration of sorafenib to a certain extent, but neither is particularly significant. However, HCC cells forming intrahepatic tumors in the liver did not have a significant effect on sorafenib blood levels. These result were in line with our expectation: after all, the metabolism of sorafenib is a function of the whole body of nude mice, and the blood concentration of sorafenib is affected by the overall function of nude mice. A single subcutaneous tumors or intrahepatic tumor tissues is not and cannot be a decisive factor in the blood concentration of sorafenib. The formation of tumor tissue in the liver, which directly affects liver function, adds to the complexity of the situation.

Although it is generally believed that the antitumor drugs for HCC are all molecularly targeted drugs represented by TKIs, it is also generally believed that HCC cells show multidrug-resistance to various cytotoxic chemotherapy drugs, some special technical methods, such as transarterial chemoembolization (TACE), can achieve the same usefulness as some cytotoxic chemotherapies for HCC treatment [53–55]. To this end, we also used four cytotoxic chemotherapy drugs while concurrently using four TKIs. TPX2 can not only induce resistance of HCC cells to the four TKIs but also induce resistance of HCC cells to the four cytotoxic chemotherapy drugs. Furthermore, the early molecularly targeted drugs were only TKIs, and in recent years, various programmed death protein 1/ programmed death ligand 1 (PD-1/PD-L1)-related research has been vigorously developed, and related drugs (checkpoint inhibitors [ICIs]) have been widely used in clinical practice [56]. Unlike TKIs, resistance to these PD-1/PD-L1 inhibitors has not been adequately studied, and the possible molecular mechanisms of resistance remain unclear [56]. Existing ICIs are mainly divided into two categories: (1) therapeutic monoclonal antibodies and (2) small molecule inhibitors [56, 57]. Among these drugs, the metabolism and clearance of small molecule inhibitors also depend on the activity of PXR in cells, so TPX2 and PXR related to this study may also affect the resistance of some ICIs.

Previously, the results from studies by Heidebrecht et al. and Vernos et al. indicated that TPX2 could be considered a cell cycle and microtubule-related protein with rough 100 kDa molecular weight [58]. TPX2 functions as a microtubule (MT)-associated protein (MAP) to modulate chromosomal instability, centrosome amplification, and the proliferation of human cancer cells. A significant amount of evidence indicates that TPX2 is an important pro-oncogene, and high levels of TPX2 have been correlated with various human malignancies (especially solid tumors) and identified as a negative factor on the prognosis of lung, liver, colon. pancreatic, breast, and cervical cancers [59–66]. Recently, a noncentrosomal TPX2 function has attracted researchers’ attention [67]. The Aurora kinase and TPX2 are co-overexpressed in several types of tumors and function as a complex [98]. The interaction between TPX2 and Aurora is essential for the proliferation of human cancerous cells [68]. TPX2 also protects Aurora from the degradation by the cdh1-activated anaphase-promoting complex/cyclosome (APC/C) proteasome’s activation [69, 70]. TPX2 was also found to promote proliferation and migration of human cancerous cells via the polo-like kinase 1 (PLK1) cascades [71, 72]. Mechanically, TPX2 mediates prostate cancer EMT through cyclin-dependent kinase (CDK1)-regulated phosphorylation of ERK/GSK3β/SNAIL pathway [73]. The results of this study show that TPX2 can function as a co-activator of PXR, which is consistent with some previous studies; Zhou et al. found that TPX2 can act as a novel co-activator of ETS-1, and Sun et al. found that TPX2 can promote endocrine dependence by facilitating the upregulation of AR activity and proliferation of prostate cancer cells [17, 18]. It is worth mentioning that the nucleo-cytoplasmic migration of nuclear receptors and transcription factors represented by PXR is necessary for its normal physiological functions [74, 75]. MTs are an important part of the cytoskeleton and form the basis for the transport of substances and subcellular components in cells [17, 18]. As an MT-associated protein, TPX2 can affect the function of MTs via an interaction with tubulin [17, 18]. Zhou et al. and Sun et al. used nucleocytoplasmic separation technology to clearly observe that TPX2 can promote the nucleocytoplasmic migration of ETS-1 or AR [17, 18]. The results of this study concerning the role of TPX2 were also confirmed by paclitaxel (which promotes microtubule aggregation) and vincristine, which promotes microtubule depolymerization [76–78]. Paclitaxel can lead to up-regulation of the effects of TPX2 on PXR recruitment to its downstream gene *cyp3a4’s* promoter and enhancer regions. Vincristine can basically block the effect of TPX2 on the recruitment of PXR on these same regions.

### Rights and permissions

Open Access This article is licensed under a Creative Commons Attribution 4.0 International License, which permits use, sharing, adaptation, distribution and reproduction in any medium or format, as long as you give appropriate credit to the original author(s) and the source, provide a link to the Creative Commons license, and indicate if changes were made. The images or other third-party material in this article are included in the article’s Creative Commons license, unless indicated otherwise in a credit line to the material. If material is not included in the article’s Creative Commons license and your intended use is not permitted by statutory regulation or exceeds the permitted use, you will need to obtain permission directly from the copyright holder. To view a copy of this license, visit http://creativecommons.org/licenses/by/4.0/.

## Supplementary information

aj-checklist

Supplemental Figure Legend

Supplemental Table 1

Supplemental Table 2

Supplemental Figure 1

## Data Availability

All data generated or analyzed during this study are available from the corresponding author on reasonable request.
