# Supplementary material for: TPX2 enhances the transcription factor activation of PXR and enhances the resistance of hepatocellular carcinoma cells to antitumor drugs
Source: Cell Death Dis. 2023 Jan 27;14(1):64. doi: 10.1038/s41419-022-05537-7 (PMC9883482; doi:10.1038/s41419-022-05537-7)
Supplement: Supplementary file 2 — Supplemental Figure Legend [file 41419_2022_5537_MOESM2_ESM.doc]

Supplemental Figure 1 TPX2 enhances the mRNA level of PXR’s downstream genes cyp3a4 or mdr-1 and the recruitment of PXR to cyp3a4’s promoter or enhancer in LS180 cells.

(A and B) The LS180 cells were transfected with control, TPX2 or siTPX2. Cells were treated with solvent control or rifampicin. The mRNA level of *cyp3a4* (A) or *mdr-1* (B) was examined using qPCR. In chromatin co-immunoprecipitation (ChIP), XREM (A) and PXRE (B) sequences were amplified respectively. The LS180 cells were transfected with control, TPX2 or siTPX2. Cells were treated with solvent control or rifampicin and analyzed using ChIP. P<0.05
