# Supplementary material for: TPX2 enhances the transcription factor activation of PXR and enhances the resistance of hepatocellular carcinoma cells to antitumor drugs
Source: Cell Death Dis. 2023 Jan 27;14(1):64. doi: 10.1038/s41419-022-05537-7 (PMC9883482; doi:10.1038/s41419-022-05537-7)
Supplement: Supplementary file 4 — Supplemental Table 2 [file 41419_2022_5537_MOESM4_ESM.doc]

Supplemental Table 2 The effect of HCC tumors (TPX2 knockdown in MHCC97-H) in nude mice on the concentration of sorafenib in nude mice’s plasma

| Time point (h) | Control | siTPX2 | Control | siTPX2 |
| --- | --- | --- | --- | --- |
|  | Subcutaneous | | intrahepatic | |
|  | concentration in plasma (ng/ml) | | | |
| 2 | 23.34±16.61 | 33.25±19.36 | 22.47±11.78 | 24.26±14.17 |
| 8 | 152.36±25.39 | 170.34±32.28 | 145.92±28.70 | 151.80±22.06 |
| 20 | 122.97±23.63 | 141.57±21.42 | 120.37±31.98 | 127.23±31.20 |
| 40 | 62.98±14.48 | 77.51±23.51 | 46.75±31.89 | 57.77±27.62 |

Table notes: After knockdown of TPX2 in MHCC97-H cells, the cells were inoculated with nude mice to form subcutaneous tumour tissue (the volume of tumour tissue was approximately 1 g) and then given 2 mg/kg via oral administration. After knockdown TPX2 in MHCC97-H cells, the cells were inoculated into nude mice to form subcutaneous tumour tissue and then in situ liver tumour tissue was established. 2mg/kg via oral administration was given to nude mice. Thereafter, blood levels of sorafenib were measured. Blood was taken from the orbital plexus at time points 2, 8, 20 and 40h (1, approximately 100μl of blood at each time point) and sorafenib levels were measured using LS-MS/MS.
