# Supplementary figures and images for: TPX2 enhances the transcription factor activation of PXR and enhances the resistance of hepatocellular carcinoma cells to antitumor drugs
Source: Cell Death Dis. 2023 Jan 27;14(1):64. doi: 10.1038/s41419-022-05537-7 (PMC9883482; doi:10.1038/s41419-022-05537-7)

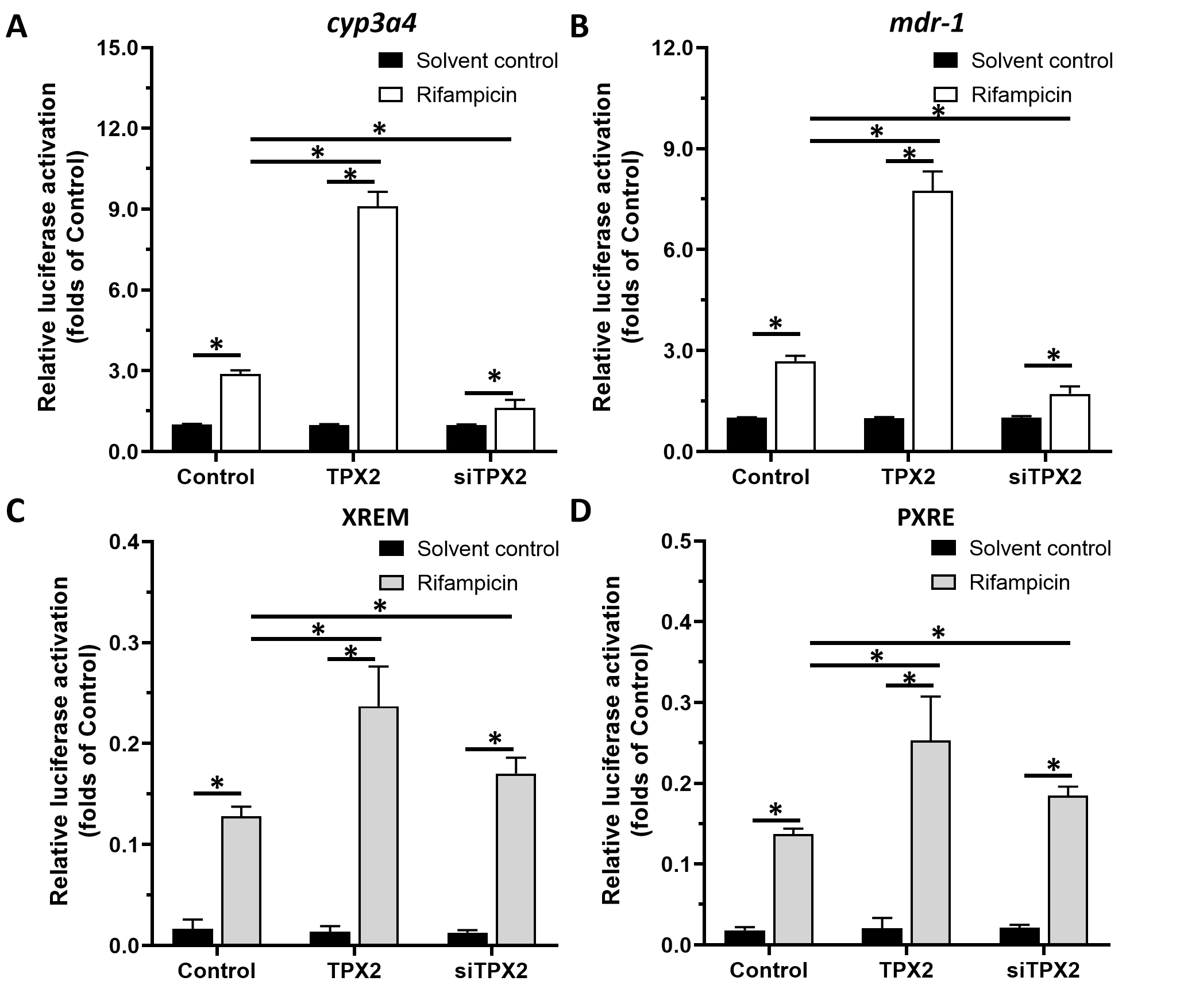

Supplement: Supplementary file 5 — Supplemental Figure 1 [file 41419_2022_5537_MOESM5_ESM.jpg]
